# Microstructural heterogeneity in the electrodeposited Ni: insights from growth modes

**DOI:** 10.1038/s41598-020-62565-z

**Published:** 2020-03-26

**Authors:** Isao Matsui, Atsuya Watanabe, Yorinobu Takigawa, Naoki Omura, Takahisa Yamamoto

**Affiliations:** 1 Structural Materials Research Institute, National Institute of Advanced Industrial Science and Technology (AIST), 2266-98 Shimo-Shidami, Moriyama-ku, Nagoya, 463-8560 Japan; 2 Department of Materials Science, Osaka Prefecture University, 1-1, Gakuen-cho, Sakai, Osaka, 599-8531 Japan; 3 Department of Materials Design Innovation Engine, Nagoya University, Furo-cho, Chikusa-ku, Nagoya, 464–8603 Japan

**Keywords:** Synthesis and processing, Materials science, Metals and alloys

## Abstract

Microstructures of electrodeposited Ni were studied from the perspective of growth modes during electrodeposition. The electrodeposited Ni had a heterogeneous microstructure composed of nanocrystalline- and microcrystalline-grains. Electron backscatter diffraction analyses showed that nanocrystalline- and microcrystalline-grains were preferentially oriented to specific planes. Secondary ion mass spectrometry also revealed that coarse-grained regions had higher S content than that of finer-grained regions. Hence, microstructural heterogeneity in electrodeposited Ni is reflected by the overlap of inhibited and free growth modes. Our discussion surrounding microstructural heterogeneity also provides insight into other electrodeposited nanocrystalline systems.

## Introduction

Nanocrystalline materials often exhibit more favorable properties than coarse-grained materials because of their nano-grain structure^1–3^. Electrodeposition is a typical process for fabricating nano-grain structures^4^. In practice, Ni and its alloys are electrodeposited with grain sizes below 100 nm^5–8^, and these electrodeposited nanocrystalline metals and alloys show high strength and good ductility^9–13^. However, electrodeposited materials have fiber-like textures, which reflect the preferred crystallographic orientation of their crystallites along the growth direction^14,15^. The texture formation of electrodeposits is determined by the electrodeposition conditions^16–18^. Amblard *et al*.^19^ explained that electrodeposition conditions change the growth mode during electrodeposition. Furthermore, they showed three types of growth modes, namely free-lateral growth, inhibited-lateral growth, and inhibited-out growth, and indicated that each growth mode resulted in specifically oriented textures on electrodeposited Ni. In the few cases where growth modes (or texture) have been considered^12,20,21^, these features have been shown to profoundly affect the microstructure and mechanical properties. Godon *et al*.^20^ reported the relationship between grain orientation and the Hall–Petch relationship in electrodeposited Ni. The Hall–Petch plot of hardness was classified into three regions based on specific textures. In a subsequent report^21^, they found that grain refinement and changes of textures were linked with an increase in impurity content. The incorporation of impurities resulted from the inhibited growth mode. Matsui *et al*.^12^ found a relationship between the tensile elongation and orientation index for the (200) plane in electrodeposited nanocrystalline Ni–W alloys. This relationship indicates that the free-lateral growth mode is suitable for producing ductile electrodeposited nanocrystalline materials.

In previous studies^12,20,21^, the effects of growth modes have been discussed by comparison of typical samples having different dominant orientations. These comparisons are based on the assumption that typical samples are electrodeposited by a single growth mode. Contrary to this assumption, our belief is that the actual electrodeposited samples form with multiple competing growth modes. Hence, the microstructure of electrodeposited samples is uniform, and a heterogeneous grain structure forms from different growth modes. The main aim of this study is to reveal the microstructural heterogeneity in electrodeposited metals and alloys. We prepared an ideal model sample of electrodeposited Ni with a heterogeneous microstructure composed of nanocrystalline- and microcrystalline-grains. The heterogeneous microstructure was characterized by electron backscatter diffraction (EBSD) and secondary ion mass spectrometry (SIMS) to verify whether the heterogeneous structure was attributed to the growth mode.

## Results

**Figure 1:**
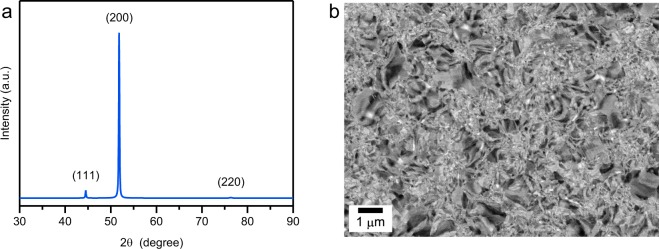
(**a**) XRD patterns of electrodeposited Ni. Electrodeposited Ni shows a strong (200) fiber texture. (**b**) Representative BSE-SEM image showing microstructure of electrodeposited Ni as-deposited state and heterogeneous microstructure consisting of nanocrystalline and microcrystalline grain regions.

The X-ray diffraction (XRD) pattern of electrodeposited Ni is shown in Fig. 1a. The XRD pattern contains the first three reflections of the face-centered cubic Ni. The electrodeposited Ni for this study had weak (111) and strong (200) peaks, which are consistent with previous reports^5,15,22^. These peaks corresponded to inhibited lateral growth and free lateral growth, respectively, according to relationship proposed by Amblard *et al*.^19^. A backscattered electron (BSE) scanning electron microscope (SEM) image of electrodeposited Ni, is shown in Fig. 1b. The heterogeneous microstructure consisted of nanocrystalline and microcrystalline grains. The grains of electrodeposited Ni were distributed over a very broad range of sizes.

**Figure 2:**
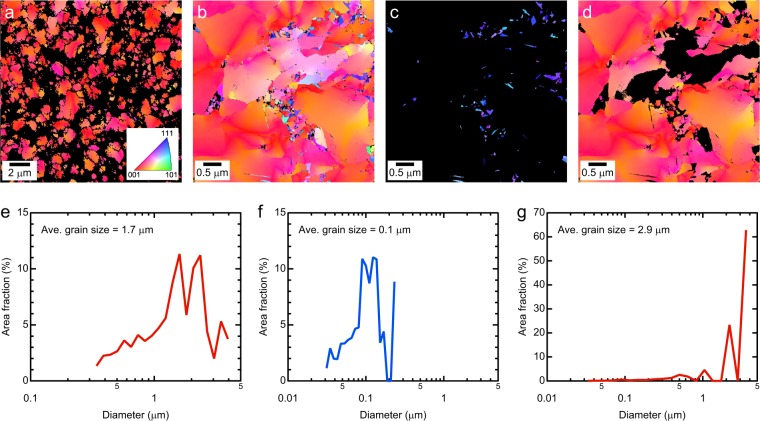
(Upper) IPF maps obtained by EBSD and t-EBSD for electrodeposited Ni and (lower) corresponding size distribution of the grains. Inset in (**a**) shows the color code. The grain-detection angle was 15°. (**a**) EBSD analysis indicates that coarse-grains have a preferred orientation along the [100] direction. (**b**) t-EBSD techniques captures nanocrystalline grains and provides information on the crystal orientation. High-resolution IPF map showing grains with an orientation within 20° along the (**c**) [111] and (**d**) [100] directions, respectively. (**e**), (**f**), and (**g**) are the size distributions corresponding to (**a**), (**c**), and (**d**), respectively.

To discuss the growth of these grains, we analyzed the crystal orientations of coarse- and fine-grain regions by EBSD and transmission-electron backscatter diffraction (t-EBSD), respectively (Fig. 2). Inverse pole figure (IPF) maps obtained by EBSD show the microstructure of electrodeposited Ni and the corresponding distributions of grain sizes are shown in Fig. 2a,e, respectively. The grains were detected at a grain detection angle of 15°. In EBSD analysis (Fig. 2a,e), grains with sizes of less than 0.32 μm are not shown, which limits the data of the coarse-grain region and remove noise of only a few pixels. Figure 2a shows the preferred orientation along the <100> direction for coarse-grained regions. The average size of these coarse-grains was 1.7 μm (Fig. 2e). Furthermore, the fraction occupied by the coarse-grains (colored) region in Fig. 2a was estimated to be 57%. Figure 2b shows a high-resolution IPF map obtained by t-EBSD for electrodeposited Ni. The t-EBSD measurements clearly captured the nanocrystalline grains and provide information on the crystal orientation. Unlike the coarse-grain region (Fig. 2a), grains with orientations along the <111> direction were confirmed in the finer-grain region. To clarify the characteristics of grains and their crystal orientations, grains with orientation within 20° of the <111> and <100> directions were selected and are shown in Fig. 2c,d, respectively. The corresponding distributions of grain sizes are shown in Fig. 2f,g. Note that grains less than 30 nm in size are not shown in the t-EBSD analysis (Fig. 2b,d,f,g) because these data were noisy. The sizes of grains with orientations along the <111> direction ranged from 0.03 to 0.23 μm, and the average size was 0.11 μm. In contrast, the sizes of grains with orientations along the <100> direction ranged from 0.03 to 3.59 μm, and the average size was 2.85 μm. The grain size varied greatly depending on the crystal orientation: inhibited lateral growth resulted in finer-grains, whereas free lateral growth resulted in coarser-grains. These results agree with those previously reported^20,21,23^.

**Figure 3:**
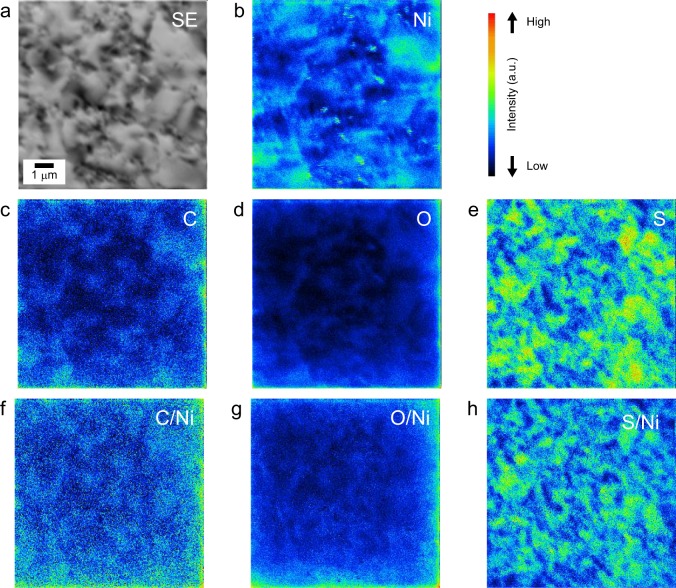
SIMS isotopic images and derived ratio of electrodeposited Ni with heterogeneous microstructure: (**a**) secondary electron, (**b**) Ni, (**c**) C, (**d**) O, and (**e**) S. (**a**) Sputtering to remove the contamination layer resulted in irregularities in the sample. (**b**) Unevenness caused a change in the intensity of the Ni matrix. (**c–e**) C, O, and S distributions were also influenced. Thus, secondary ion images of (**f**) C, (**g**) O, and (**h**) S were normalized according to the Ni content.

To reveal the impurity distribution in the heterogeneous microstructure, we applied NanoSIMS analysis, as shown in Fig. 3. The secondary electron (SE) image after sputtering to remove surface contamination (Fig. 3a), shows that sputtering introduced irregularities onto the surface. The formation of irregularities by the ion beam is confirmed in past reports^24,25^, and is more remarkable in a polycrystal than in a single crystal^26^. This is caused by variation of the sputtering rate depending on the crystal orientation. The black and gray parts correspond to finer- and coarser-grain regions, respectively. The irregularities on the surface also changed the intensity of the Ni matrix. As shown in Fig. 3b. C, O, and S (Fig. 3c,e) were also under the same influence. Thus, the secondary ion images of C, O, and S were normalized by Ni intensity and the normalized images are shown in Fig. 3f,h, respectively. Unfortunately, C and O contents were low in content (~10 ppm) and difficult to separate from the background (Fig. 3f,g). Figure 3h indicates that S was inhomogeneously distributed on the mesoscale of the electrodeposited Ni. Highly intense signals from S correspond to coarser-grains. Notably, S is an impurity derived from addition of saccharin^5^, and these results indicate that adsorption of saccharin occurred during electrodeposition of coarse grains.

## Discussion

**Figure 4:**
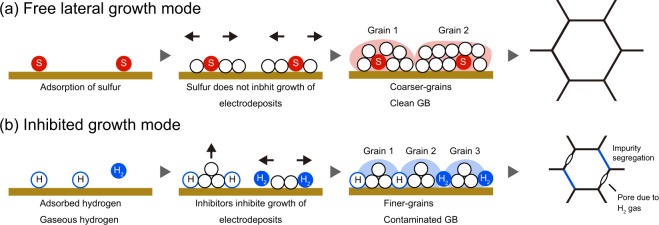
Schematic illustration showing how microstructures formed during electrodeposition with (**a**) free lateral growth mode and (**b**) inhibited growth mode.

Amblard *et al*.^19^ described free-lateral growth as a mode in which there is no inhibitor (such as hydrogen gas or adsorbed hydrogen) and no other influences on the electrodeposits. In this study, S was detected in coarse-grains oriented along the <100> direction, which is considered to be formed from free-lateral growth, as shown in Fig. 3. Hence, S adsorption promoted free-lateral growth. Cases in which free-lateral growth was promoted by additives and the solute have been reported in the literatures^27^. If S inhibited Ni growth, in the same way that hydrogen gas or adsorbed hydrogen, the addition of saccharin might facilitate the inhibited growth mode and decrease the grain size. Thus, S is unlikely to inhibit Ni growth. Rather, the adsorption of S on the growth surface may suppress the adsorption of hydrogen and hinder the inhibited growth mode. This assumption is consistent with the fact that S does not segregate at grain boundaries (GBs) and exists within the grains, as shown in Figs. 3a,h. Schematic images of the above growth mechanism are summarized in Fig. 4a.

Compared with free-lateral growth, inhibited lateral growth resulted in finer grains, as shown in Fig. 2c,f. This trend has also been reported in the past studies^21,23^. The grain refinement is attributed to an increase in the impurity content. In fact, in electrodeposited Ni and Ni alloys, correlations have been confirmed between impurity^22^ and solute contents^7,8^, and the grain size. Impurities and solute segregation have been proposed as the primary stabilizing mechanism of nanocrystalline structures^28,29^: the segregation reduces the GB energy and minimizes the thermodynamic driving force for grain growth. From these results, we assume that the incorporated impurities such as hydrogen gas or adsorbed hydrogen were present on the GBs in the inhibited growth modes. Hence, the inhibitor inhibited growth of electrodeposits and promoted formation of GBs. Schematic images of the inhibited growth mode are also shown in Fig. 4b. In Fig. 4b, hydrogen is shown as an inhibitor, but this does not limit the inhibitor to only hydrogen^21^.

The t-EBSD captured nanocrystalline grains with orientations along with the <100> texture, which formed by the free-lateral growth modes, as shown in Fig. 2d,g. These grains maintained their grain size by being surrounded by grains formed through inhibited growth modes. In the case shown in Fig. 4, there are three kinds of GBs, namely coarse-coarse grains, fine-fine grains, and coarse-fine grains. The GBs between the coarse grains and the fine grains would be at a medium contamination level. In fact, in study^30^ in which GB segregation of electrodeposited alloys was analyzed by atom probe tomography, the concentration of impurities at the GB varied greatly depending on the location. We suggest that a combination of the crystal orientation analysis (growth mode) and atomic-scale analysis performed in the previous research^30^ will provide a deeper understanding. At the same time, for example, the bulk H concentration of electrodeposited Ni for this study was only 2 ppm. It is also a technical challenge to be able to accurately detect trace light elements.

We derived the model, as shown in Fig. 4, to explain the connection between growth modes and the microstructure of electrodeposited material: a free growth mode introduced coarser-grains with clean GBs, whereas inhibited growth modes introduced finer-grains with contaminated GBs. In practice, this model is in good agreement with the grain growth behavior of electrodeposited materials. For Ni-based electrodeposits, abnormal grain growth during the heat treatments is a common phenomenon^31,32^. EBSD analyses of the electrodeposited Ni and Ni alloys after abnormal grain growth, showed that the microstructure orientated along the <111> directions^33–35^. Many abnormal grain growth theories have been proposed^36–38^. Although there is no general model to describe abnormal grain growth, in most mechanisms, anisotropic GB features are commonly believed to result in preferential growth. Gaseous hydrogen as an inhibitor is incorporated into the growth of the electrodeposits, and pores are generated in the grains and at GBs^39,40^. These GB features promote abnormal grain growth. In addition, the driving force of abnormal grain growth is the excess free energy of GBs Δ*G*_GB_, as given by:

1 $$\Delta {G}_{GB}=2\sigma V/d,$$

where *σ* is the GB energy, *V* is the molar volume, and *d* is the grain size. The driving force increases as the grain size decreases. As described above, grains formed via the inhibited growth mode are finer and GBs are contaminated. These features are favorable for abnormal grain growth. Therefore, crystal grains oriented along the <111> directions via inhibited-lateral growth become seeds that cause abnormal grain growth, resulting in texture evolution^33–35^. The model developed in this study (Fig. 4) might explain the behavior of other electrodeposited systems. Thus, electrodeposited materials have a microstructural heterogeneity reflecting the growth mode in the same manner as electrodeposited Ni for this study. The cause of the competition between the growth modes at the cathode would be local bias such as current or bath composition, and the effects should be considered more fully. The discussion of the heterogeneity in terms of meso-scale of electrodeposits gives insight into electrodeposited nanocrystalline metals and alloys. This study also points out the need to consider heterogeneity when performing high-resolution analyzes such as atom probe tomography and aberration-corrected scanning transmission electron microscopy on electrodeposited materials, because the application area of these analyzes is very local.

## Conclusions

Electrodeposited Ni with a heterogeneous microstructure composed of nanocrystalline- and microcrystalline-grains was characterized by EBSD and SIMS techniques. The combined use of EBSD and t-EBSD revealed the crystallographic orientation of the heterogeneous microstructure: coarse grains were orientated along the <100> direction, whereas fine grains were oriented along the <111> direction. Furthermore, NanoSIMS measurements indicated that S was present in the coarse-grain regions. Our discussion, based on these results, indicates that S promoted free-lateral growth by suppressing adsorption of inhibitors. Because S does not segregate at GBs, coarse-grains were formed. Furthermore, our discussion points to the possibility that inhibited growth modes cause impurities to segregate at GBs. This study indicates the possibility that electrodeposited materials have a heterogeneous microstructure reflecting the growth modes. Thus, it is important to elucidate the mechanism and characteristics based on the mesoscale heterogeneity of electrodeposits.

## Methods

### Electrodeposition

Bulk samples of Ni, with length of 50 mm, width of 30 mm, and thickness of ~1.2 mm, were prepared by electrodeposition. The deposition bath was composed of 300.0 g/L nickel sulfate tetrahydrate, 5.0 g/L nickel chloride hexahydrate, 20.0 g/L sodium propionate, 4.2 g/L sodium gluconate, 1.0 g/L sodium saccharin dihydrate, and 0.3 g/L sodium lauryl sulfate. All samples were deposited onto copper substrates of commercial purity using nickel plates (99.98%). Electrodeposition was performed using 1-L deposition systems and details of the setup have been previously reported^41,42^. Electrodeposition was conducted at a current density of 25 mA/cm^2^, bath temperature of 55 °C, and pH of 4.0 for 96 h. The pH value was maintained by adjustment with 1.0 mol/L amid sulfuric acid solution before and during electrodeposition. After electrodeposition, the bulk plates of electrodeposited Ni were cut into specimens for the following analyses.

### Microstructure analysis

XRD (Rigaku MiniFlex 600) analysis was conducted on the samples in the as-deposited state. The XRD equipment was operated at 15 mA and 40 kV with Cu Kα radiation. The microstructure was observed by a SEM (JEOL JSM-IT300HR) operated at a 15-kV acceleration voltage. EBSD and t-EBSD^43,44^ techniques were used to determine the grain size and characterize the crystal orientation. EBSD maps were acquired at a 15-kV acceleration voltage on a SEM (JEOL JSM-7001F) with a 0.08-μm measurement step, and t-EBSD maps were acquired at an accelerating voltage of 30 kV on a SEM (Carl ZEISS SUPRA40VP) with a 0.01-μm measurement step. For SEM observations and EBSD analyses, the electrodeposited samples were mechanically polished with SiC paper and then mirror-finished with diamond and colloidal silica. For t-EBSD analyses, the thin foils were prepared by a conventional twin-jet electropolishing technique. The electrodeposited samples were cut into disk shapes with a diameter of 3 mm by a punching apparatus. The cut disks were then ground, polished, and completed by a twin-jet electropolishing. The electropolishing was performed as previously reported^12,45^. The C and S contents, which are major impurities for Ni-based electrodeposits^46^, were quantified by IR absorption after combustion in a high-frequency induction furnace (LECO CS844). The C and S contents were determined to be 10 ppm (0.01 at%) and 200 ppm (0.04 at%), respectively. SIMS (CAMECA NanoSIMS 50 L) was also used to clarify the distribution state of both impurities in the electrodeposited Ni. SIMS measurements were performed with the use of a Cs^+^ beam and an impact energy of 16 keV. In addition, H content, which is related to the major inhibitors, such as gaseous hydrogen and adsorbed hydrogen, was quantified by inert gas fusion thermal conductivity detection (LECO RHEN602). The H content was determined to be 2 ppm.

## Data Availability

The data that support the findings of this study are available from the corresponding author on reasonable request.
